# Primary care provider perspectives on a planned multi-cancer early detection test clinical trial

**DOI:** 10.1016/j.conctc.2023.101183

**Published:** 2023-07-03

**Authors:** Ronald Myers, Mie H. Hallman, Kaitlyn Davis, Melissa DiCarlo, Constantine Daskalakis, Ayako Shimada, Christopher Chambers

**Affiliations:** a Department of Medical Oncology, Thomas Jefferson University, 834 Chestnut Street, The Franklin Building, Suite 314, Philadelphia, PA, 19107, USA; b Department of Family and Community Medicine, Thomas Jefferson University, 1015 Walnut St., Suite 401, Philadelphia, PA, 19107, USA; c Department of Pharmacology and Experimental Therapeutics, Thomas Jefferson University, 1015 Chestnut Street, Suite 520, Philadelphia, PA, 19107, USA

**Keywords:** Multi-cancer early detection, Cancer screening, Primary care, Primary care providers

## 1 Introduction

Randomized clinical trials of multi-cancer early detection (MCED) tests are being conducted and more are being planned [1,2]. The support of primary care providers (PCPs) for these trials will be needed to ensure that screening-eligible patients from diverse populations are recruited [3]. Little is known, however, about PCP support for patient participation in MCED trials [4]. To our knowledge, the current study is the first to assess PCP support for patient participation in research on MCED testing.

## 2 Methods

The research team identified four primary care practices (two family medicine and two internal medicine practices) in Jefferson Health, a large, urban health system in Philadelphia. The team recruited PCPs (physicians and nurse practitioners) from the practices and provided them with a link to a patient-oriented infographic that provided details about a planned MCED trial for persons who were 50–80 years of age.

The infographic explained that in a multi-year trial, health system research coordinators would be responsible for recruiting and following patients in the MCED trial. In addition, the infographic noted that participants would undergo serial blood draws for MCED testing and would be randomly assigned to either a control group or an intervention group. The control group would receive usual care, with retrospective MCED test analysis. The intervention group would receive usual care, with immediate MCED test analysis and follow-up, as needed. The description of the trial also stated that costs associated with testing and follow-up would be covered for both groups. After viewing the infographic, PCPs were asked to complete a brief, self-directed online survey hosted on Qualtrics^XM^.

The survey described the trial and included a single item that asked respondents if they intended to support patient participation in the MCED trial, with the following response options: “Yes”, “No”, or “Unsure.” Providers were given the opportunity to report the main reason for their answer as an open-ended response. In addition, the survey included a section with statements that reflected factors that could affect PCP support (positively or negatively) for patient participation in the trial. Responses to these statements used a 5-point Likert type response set that ranged from 1 = Strongly Disagree to 5 = Strongly Agree. Finally, the survey elicited information about background characteristics.

This research project was part of a larger study conducted to ascertain the feasibility of engaging PCPs and of enrolling primary care patients in a planned MCED trial. The study was approved by Thomas Jefferson University's Institutional Review Board (IRB #21C.806).

## 3 Results

We identified 37 PCPs in the practices, and 27 (73%) completed the survey. About half of the respondents were women and two thirds were white; 81% were physicians while 19% were nurse practitioners, 54% were in practice for less than 20 years, all were board certified, and 74% had a hospital affiliation (Table 1).

**Table 1:** Background characteristics (n = 27).

Variable	n (%)
**Age (years)**^a^	
<40	5 (19)
40-59	13 (50)
60+	8 (31)
**Sex**^a^	
Female	13 (50)
Male	13 (50)
**Race/Ethnicity**^a^	
White	16 (62)
African American	1 [4]
Latino/Hispanic	3 (12)
Asian	4 (15)
Other	2 (8)
**Provider**	
Physician	22 (81)
Nurse Practitioner	5 (19)
**Years since MD/NP degree**^a^	
<10	5 (19)
10-19	9 (35)
20+	12 (46)
**Board Certified**	
Yes	27 (100)
No	0 (0)
**Hospital affiliation**	
Yes	20 (74)
No	7 (26)
**Hospital admitting privileges**	
Yes	18 (67)
No	9 (33)

a Data missing for one respondent.

Table 2 shows that responses regarding intention to support patient participation was as follows: Yes: 25 (93%), No: 1 (4%), and Unsure: 1 (4%). There were 21 respondents who provided a reason for their response. The most commonly reported reasons for supporting patient participation were the belief that early cancer detection is important, and scientific research could help to develop new cancer screening tests. Respondents who indicated that they did not support or were unsure about supporting patient participation reported concerns about how to manage false positive results among trial participants, and staff burden related to patient needs.

**Table 2:** Intention to support patient participation in the MCED clinical trial (n = 27).

Variable	n (%)
**If the MCED trial were open today, would you support patient participation in the trial?**	
Yes	25 (92)
Unsure	1 (4)
No	1 (4)

The complete set of responses shown in Table 3 provides a more detailed picture of provider perspectives about the trial. The data indicate that PCP intention to support patient participation in the MCED trial was moderately high. The overall score (13 items, 5 reverse-coded, alpha = 0.83) had a range of 1.9–4.6, mean of 3.8, and standard deviation of 0.6. Inspection of responses to individual survey items provides more insight into factors that explain provider intention to support patient participation in the MCED trial.

**Table 3:** Factors affecting provider intention to support patient participation in the MCED clinical trial (n = 27).

Variable	Strongly Disagree n (%)	Mildly Disagree n (%)	Uncertain n (%)	Mildly Agree n (%)	Strongly Agree n (%)
I think that supporting the trial is an important thing for me to do	0 (0)	1 (4)	0 (0)	10 (37)	16 (59)
I believe that supporting the trial makes good sense	0 (0)	1 (4)	1 (4)	3 (11)	22 (81)
I believe that the trial will help to identify patients with early cancer	0 (0)	1(4)	2 (7)	12 (44)	12 (44)
I believe that supporting the trial will help my patients	0 (0)	1 (4)	3 (11)	11 (41)	12 (44)
I think that the trial will generate information that will improve patient outcomes	0 (0)	1(4)	3 (11)	11 (41)	12 (44)
I believe that research coordinators could help patients make decisions about joining the trial	0 (0)	1 (4)	3 (11)	10 (37)	13 (48)
I believe that other physicians would support the trial	0 (0)	0 (0)	4 (15)	13 (48)	10 (37)
I believe that trial research coordinators will provide the help I need to support the trial	1 (4)	3 (11)	4 (15)	9 (33)	10 (37)
I am concerned about managing trial participants within my own practice who are diagnosed with cancer^b^	6 (22)	8 (30)	2 (7)	10 (37)	1 (4)
I think that supporting the trial would require too many practice resources^b^	3 (11)	8 (30)	7 (26)	7 (26)	2 (7)
I believe that supporting the trial would require too much of my time and effort^a^,^b^	4 (15)	4 (15)	11 (42)	6 (23)	1 (4)
I am worried about managing patients with a positive blood test result in the trial^a^,^b^	1 (4)	4 (15)	4 (15)	11 (42)	6 (23)
I am concerned about having the time to explain the trial to patients^b^	1 (4)	2 (7)	5 (19)	14 (52)	5 (19)

a Data missing for one respondent.

b Response was reverse coded for computation of mean score and range for factors affecting provider intention.

The most common factors favoring support for the trial were the respondents’ beliefs that supporting the trial “is a good thing” (96%), “makes good sense” (93%), “will help identify patients with early cancer” (89%), and “will help my patients” (85%). Respondents also voiced some concerns, including “having the time to explain the trial to patients” (71%), “managing patients with a positive blood test result” (65%), and “managing patients within my own practice who are diagnosed with cancer” (41%).

## 4 Discussion

Results of this study indicate that over 90% of PCPs in the study intended to support patient participation in a hypothetical MCED trial. This finding is consistent with findings reported by Getz in a study that involved administering a survey to physicians and nurses to assess their interest in referring patients to clinical trials.[5] That report, which focused on clinical trials in general, not MCED trials, found that 72% of physicians and 69% of nurses were interested in referring patients to trials.

Further exploration of factors that could account for PCP intention to support patient participation in an MCED trial revealed that providers believed in the value of research and the potential benefits that trial participation could have for their patients. In a recent report of focus group research with primary care providers, Taft et al. also found that support for clinical trials in general was associated with the belief that such research was of scientific value and could benefit patients.[6] They also reported that providers in their study felt a need to protect their patients’ well-being and expressed concern about the management of patients who would be identified as trial candidates.

We used an infographic designed for patients to describe the trial to providers. In addition, we explained to providers that health system research coordinators would help to recruit and guide patients through trial participation. This aspect of trial design may have alleviated some concerns among survey respondents about the time and effort that otherwise might be associated with recruiting study participants. In addition, survey respondents tended to believe that other providers were likely to support patient participation in an MCED trial, highlighting the potential importance of social support and influence.

It is important to note that even though the role of research coordinators in patient recruitment was explained, a substantial number of providers reported that they were concerned about the time they might have to address patient concerns that may arise during the trial, including the management of patients who would have a positive MCED test result and those who would be diagnosed with cancer in the MCED trial. These concerns suggest that as part of the process of preparing PCPs for the launch of any new MCED trial, health system leaders should educate providers about the trial design, clarify their role in the management of trial participants, and ensure that there are adequate resources available to support the clinical management of trial participants.[7]

This study had two main strengths: a high participation rate at 73% and the fact that it is the first survey to assess PCPs’ thoughts on an MCED clinical trial. In terms of study limitations, the number of providers who completed the survey came from four primary care practices in one health system. In addition, the number of survey respondents was small. These factors may limit the generalizability of reported findings. It is also the case that although the research team gave providers access to the patient infographic, we cannot be certain whether or how carefully survey respondents reviewed the informational content therein. Thus, we cannot ascertain the extent to which exposure to the content of the infographic may have affected provider responses.

## 5 Conclusion

The current study involved administering a survey to assess PCP support for patient participation in a planned MCED test clinical trial and identify factors likely to affect PCP support. We determined that the overwhelming majority of PCPs in the study supported patient participation in an MCED test trial. We also found that although PCPs tended to believe that trial participation would help to identify patients with cancer who could benefit from early intervention, they expressed concerns related to management of trial participants. It is important to note that PCP support will be needed to achieve recruitment and adherence objectives of future MCED clinical trials being planned by the National Cancer Institute and MCED test developers.

To build on the current study, the research team is planning to survey PCPs across the health system in order to gain a more complete understanding of PCP support for patient participation in future MCED research. In addition, we aim to develop a robust tool that may be used to identify potential barriers and facilitators to patient recruitment from the provider perspective.[7] Ancillary to these PCP-related efforts, the research team also aims to survey primary care patients across the health system, so as to determine the interest of persons from diverse populations in joining planned MCED trials.[8] These efforts are intended to develop validated tools that can be used to gather information needed to plan the successful implementation of future MCED research trials in health systems.

## Funding

This work was supported by a grant from Exact Sciences Corporation, Madison, WI, as well as by Thomas Jefferson University's Sidney Kimmel Cancer Center [Cancer Center Support Grant 5P30CA056036-17].

## Declaration of competing interest

The authors declare the following financial interests/personal relationships which may be considered as potential competing interests: This work was supported by a grant from Exact Sciences Corporation, Madison, WI, as well as by Thomas Jefferson University's Sidney Kimmel Cancer Center [Cancer Center Support Grant 5P30CA056036-17].
